# Some factors affecting on the severity of Acute Psychoses in Alcohol Withdrawal

**DOI:** 10.1192/j.eurpsy.2022.2133

**Published:** 2022-09-01

**Authors:** V. Kuzminov

**Affiliations:** SI “Institute of neurology, psychiaatry and narcology NAMS of Ukraine”, Emergency Psychiatry And Narcology, Kharkiv, Ukraine

**Keywords:** factors of severity, Alcohol dependence, alcohol withdrawal state with delirium

## Abstract

**Introduction:**

Alcohol withdrawal delirium is severe complication of alcohol withdrawal leading to high mortality. Early identification of severe course of psychosis and complications threatening the patient’s life is the most important problem in the treatment of these patients.

**Objectives:**

Under supervision were 690 men, dependent on alcohol, in the state of withdrawal with acute psychotic disorder (primary hospitalization in the framework of this study); the average age - (39,9 ± 3,4 years), the average age of alcohol abuse - (9,7 ± 1, 1 years). The patients were examined in a dynamics after a re-hospitalization after 5-7 years. This allowed us to verify the differential diagnostic approach to acute psychotic disorders in a state of abandonment, to investigate the impact of chronic acute psychotic disorders on the course of alcohol dependence, including the formation of deficits.

**Methods:**

clinical, clinical and psychopathological, methods of quantified scales and mathematical statistics.

**Results:**

There were estimated factors influencing the severity of alcohol withdrawal with delirium: total amount of alcohol consumed per week, drunken alcoholics, persistent alcohol abuse, social disadaptation, cognitive impairment, psychological disorders, reducing the quality of alcohol consumed, food pattern characterized total calorie mostly due to alcohol, life trajectory, severe or chronic somatic diseases, rate of progression of alcohol dependence.

**Conclusions:**

The severity of acute psychotic disorder in the state of alcohol withdrawal mostly depended situational factors such as the number of days of severe drinking before a psychotic disorder, the pattern of nutrition, the quality and quantity of alcohol consumed, the presence of acute somatic diseases.

**Disclosure:**

No significant relationships.

